# Dengue in northeastern Brazil: a spatial and temporal perspective

**DOI:** 10.1590/0037-8682-0435-2020

**Published:** 2020-12-11

**Authors:** Ana Beatriz Souza Martins, Francisco Gustavo Silveira Correia, Luciano Pamplona de Góes Cavalcanti, Carlos Henrique Alencar

**Affiliations:** 1 Universidade Federal do Ceará, Faculdade de Medicina, Programa de Pós-graduação em Saúde Pública, Departamento de Saúde Comunitária, Fortaleza, Ceará, Brasil.; 2 Centro Universitário Christus - (UNICHRISTUS), Fortaleza, Ceará, Brasil.

**Keywords:** Dengue, Spatial analysis, Temporal analysis

## Abstract

**INTRODUCTION:**

The state of Ceará (Brazilian Northeast) has a high incidence of dengue. Therefore, we aimed to characterize the temporal patterns and spatial distribution of dengue cases in Ceará during 2001-2019.

**METHODS:**

A spatiotemporal ecological study was performed with secondary data. Time-trend analysis was performed using a segmented log-linear regression model to estimate the average annual percentage change (AAPC) and the annual percentage change (APC) in incidence of dengue. We also performed spatiotemporal analysis to identify the place, time, and relative risk (RR) of dengue clusters.

**RESULTS:**

There were 539,653 dengue cases. The AAPC reduced over time (-9.5%; 95% confidance interval [CI]: -18.3; -0.3). Three trends were identified-2001-2004: APC=-20.9% (95% CI: -65.1 to 44.8), 2005-2015: APC=7.9% (95% CI: -6.0 to 98.9), and 2016-2019: APC=-48.8% (95% CI: -83.0 to -6.1). During 2001-2007, 10 significant clusters were identified (RR=3.57-14.38: n=4 and RR=0.05-0.39: n=6). During 2008-2013, there was 1 cluster in the western region (RR= 3.40) and four other clusters (RR=0.02-0.15). The last period presented 5 high-RR clusters (RR=2.95-9.24). The low-RR clusters were located in the central-north, central-south, south, and northwest regions. However, the central-west region remained a high-RR cluster region throughout the study period.

**CONCLUSIONS:**

Dengue showed a decreasing incidence. During the epidemic years, the southern, eastern, and western regions presented high-risk clusters. Introduction of a new dengue serotype in a low-RR area can cause explosive outbreaks due to population susceptibility.

## INTRODUCTION

Dengue is a systemic and dynamic infectious disease caused by an arbovirus of the *Flaviviridae* family, *Flavivirus* genus, which has four serotypes: DENV-1, DENV-2, DENV-3, and DENV-4^1^ ^,^ ^2^. Infection with one of these serotypes results in permanent immunity against that serotype and temporary immunity against the other three serotypes^3^.

In Brazil, dengue considerably influences losses of healthy life years as it affects numerous people and causes some degree of disability during infection and considerable case fatality in children^4^. Brazil has the highest economic cost (42%) for dengue in America, representing 42% of the total value^5^. In 2019, Brazil accounted 70% of the 3 million registered cases in the American continent, with an incidence of 1,038/100 thousand inhabitants^6^.

The state of Ceará, located in the Northeast region of Brazil, has reported a high incidence of dengue in successive epidemics since 1986^7^ ^-^ ^9^. This is because Ceará has areas that have favorable environmental conditions such as high temperatures and high humidity and municipalities with low socioeconomic conditions and highly frequent water crises. In addition, the state is a tourist hub with an intense flow of visitors, thereby increasing the risk of disease entry and circulation^7^ ^-^ ^9^.

Dengue transmission results from a stable relationship between the virus, vector, human being, and geographical space. Regarding geographic space, there are factors that contribute to a higher risk of dengue and also contribute to its heterogeneous distribution^10^. Epidemiology not only analyzes the distribution of diseases and their determinants in the population, time, and space but also unveils factors that influence the health-disease process^11^.

Spatial analysis is an important tool for health risk analysis, especially when it is associated with the population’s socioeconomic profile^12^. In contrast, temporal analysis identifies non-random patterns and effects of external factors on disease distribution in a longitudinal series^13^. These allied techniques allow us to predict the occurrence of a disease in the short or long term and evaluate the epidemiological impact of interventions.

A spatial and temporal analysis of dengue in Brazil identified that epidemics had a cyclical pattern from 1990 to 2017, with interepidemic periods of 3 or 4 years; however, these interepidemic periods have decreased over the years. The Northeast region is a region with high incidence rates and records of successive epidemics. Since 2016, states such as Ceará, Rio Grande do Norte, and Bahia have had incidence coefficients >1,500 cases/100 thousand inhabitants. In addition, Pernambuco and Alagoas recorded the highest proportions of deaths related to severe dengue^14^. Moreover, the Northeast region of Brazil was identified as the second region with the highest number of confirmed cases in Brazil^15^.

Dengue has a 34-year-long history in Ceará, and between epidemics and non-epidemic years, dengue has become an established endemic disease. Few studies in Ceará have analyzed the temporal trend and where there are risk areas for dengue in Ceará. Hence, this study aimed to characterize the patterns of temporal and spatial distribution of dengue cases in Ceará from 2001 to 2019.

## METHODS

An exploratory ecological study with a temporal and space-time design was performed in Ceará. The state has >9 million inhabitants, with a territorial extension of approximately 150 million km² and 184 municipalities^16^. It predominantly has a hot tropical semi-arid climate, with average temperatures around 27ºC^17^.

Information on dengue cases during 2001-2017 was extracted from the National System of Notifiable Diseases (*Sistema Nacional de Agravos de Notificação* in Portuguese), available at Department of Informatics of the Brazilian Unified Health System (*Departamento de Informática do Sistema Único de Saúde do Brasil*). For 2018 and 2019, data were obtained through epidemiological bulletins prepared by the Health Department of of Ceará. The study included confirmed cases based on laboratory and clinical-epidemiological criteria.

Population data were obtained from population censuses of 2000 and 2010 (Brazilian Institute of Geography and Statistics [*Instituto Brasileiro de Geografia e Estatística,* IBGE]). For the inter-census years, the population was obtained using the National Household Sample Survey, [*Pesquisa Nacional por Amostras de Domicílio*, IBGE] estimate.

### Time-trend analysis

Time-trend analysis was performed via log-linear segmented regression models using the Joinpoint Regression Program version 4.0.4 (United States National Cancer Institute, http://surveillance.cancer.gov/joinpoint/). The analysis performs a series of linear regressions predicting, through regression lines, the temporal trend of the observed values of the incidence coefficient year by year. This software estimates the average annual percentage change (AAPC) and the annual percentage change (APC) in the incidence of dengue and identifies inflection points. The inflection points represent changes (increase or decrease) in the incidence of dengue over time.

Joinpoint analysis uses Monte Carlo permutation tests to compare several models and evaluate the best segment to explain the trend over time. The input data for this analysis were the incidences of dengue per year in the state and the corresponding year. The parameters used were a minimum of zero, a maximum of three joinpoints, and 4,499 permutations. For this analysis, a point of change in the trend presented an error of 5% and a confidence interval of 95%. 

### Space-time analysis

SatScan software version 9.6 was used for space-time analysis. The 184 municipalities in the state of Ceará were used as the units of analysis using the geographical coordinates (latitude; longitude) of their headquarters. The alternative hypothesis was that the incidence of dengue in each evaluated area was higher than that reported in other areas^18^ ^-^ ^20^.

For each area, the likelihood ratio was calculated by Monte Carlo simulations to verify whether the circumscribed region corresponded to a cluster^18^ ^-^ ^20^. During scan analysis, the area that obtained the maximum likelihood ratio represented the cluster that was least likely to have occurred randomly^20^. The program used a likelihood ratio test to check whether the clusters of municipalities were areas with a statistically different incidence from the surrounding municipalities, with a significance of 0.05. The scanning window had a cylindrical format in the space-time method, where the base represents the space and the height represents the time^18^. In this analysis, it is possible to identify the location and time period in which the disease clusters were active.

The analysis period was divided into three groups-2001-2007, 2008-2013, and 2014-2019. The relative risks (RR) were calculated as the absolute number of dengue cases per municipality of residence per year and the population of each municipality.

The parameters were precision time per year, radius of 100 kilometers, statistical inference with 99,999 permutations, and a statistical significance of 95%. High-RR (RR>1) and low-RR (RR<1) areas for the incidence of dengue were represented on maps using ArcMap 9.2 software. Clusters formed by only one municipality were removed.

### Ethical considerations

The research was performed according to the principles of Resolution 466/2012 of the National Health Council (autonomy, non-maleficence, beneficence, justice, and equity). Secondary data were obtained without identification of the subjects and, therefore, informed consent was not applicable.

## RESULTS

### Time-trend analysis

**FIGURE 1:**
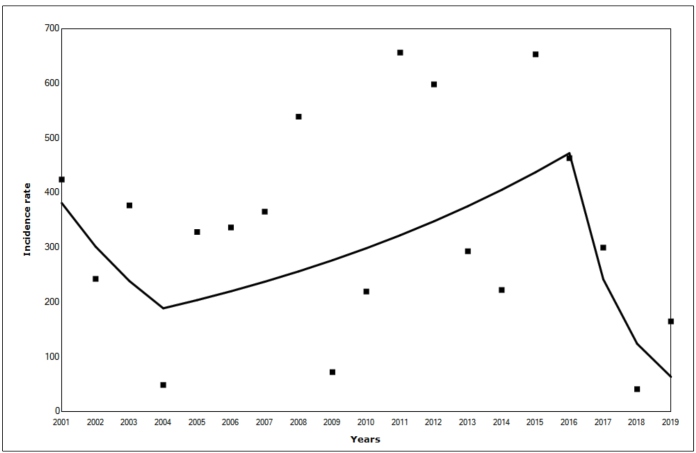
Segmented temporal trend in the incidence of dengue in Ceará from 2001 to 2019.

During 2001-2019, there were 539,653 confirmed dengue cases in Ceará. The highest number of cases were reported in 2015 (58,862), followed by 2011 (56,665). The average annual percentage change during 2001-2019 revealed a significant reduction in cases over time (AAPC=-9.5; 95% CI: -18.3 to -0.3). Two joinpoints were identified in time trend analysis; the first one in 2004 and the second one in 2016 (Figure 1). The first period (2001-2004) showed a gradual decrease in the incidence coefficient, but it was not statistically significant (APC=-20.9; 95% CI: -65.1 to 44.8). The lowest incidence of dengue in the historical series was in 2004 (48.32 cases/100 thousand inhabitants).

The first change in the trend that occurred in during 2005-2015 was an increasing trend in the incidence coefficient; however, it was not statistically significant (APC=7.9; 95% CI: -6.0 to 98.9). A high incidence rate was reported in 2008 (538.84 cases/100 thousand inhabitants), followed by 2011 (656.12 cases/100 thousand inhabitants), 2012 (597.12 cases/100 thousand inhabitants), and 2015 (652.96 cases/100 thousand inhabitants). The second joinpoint occurred during 2016-2019 (APC=-48.8; 95% CI: -83.0 to -6.1), with a significant reduction in the incidence of dengue (463.15 cases/100 thousand inhabitants in 2016 to 164.64 cases/100 thousand inhabitants in 2019). The lowest incidence rate in the time horizon was in 2018 (40 cases/100 thousand inhabitants). The points on the graph indicate the observed incidence coefficients in each year, and the lines represent the predicted coefficients in the time-trend analysis (Figure 1).

### Spatiotemporal analysis

**FIGURE 2:**
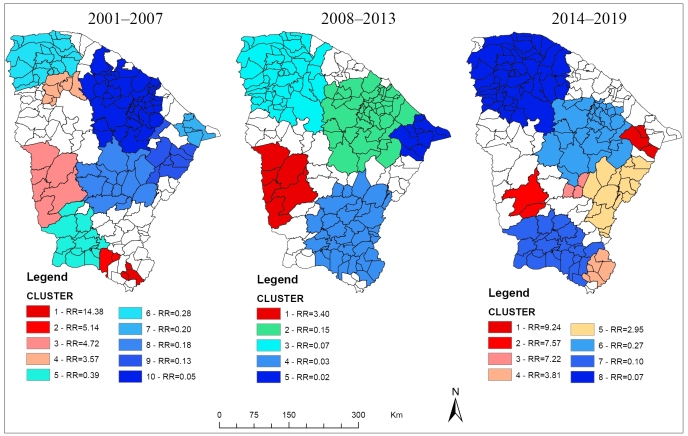
Spatiotemporal analysis of dengue cases in Ceará from 2001 to 2019. **RR:** relative risk.

During 2001-2007, 10 statistically significant clusters were identified (Table 1). Four clusters were identified with high RRs-in the extreme south of Ceará, 2007 (RR=14.38) and 2001 (RR=5.14); in the west, 2001-2003 (RR=4.72); and in the north, 2006-2007 (RR=3.57). Spatial clusters with low RRs (RR=0.05-0.39) were located in the southwest (cluster 5), east (clusters 7 and 9), north (cluster 6), central (cluster 8), and central-north regions (cluster 10) (Table 1; Figure 2A).

**TABLE 1:** Statistically significant aggregates of dengue cases in Ceará from 2001 to 2019 according to space-time analysis.

Cluster	Time	Number of	Population	Radius	Incidence/	Relative	P
		Municipalities	at risk	(Km)	100,000	risk	Value
					Inhabitants		
**2001-2007**							
1	2007	3	65,260	16.45	4,434.56	14.38	<0.00001
2	2001	2	341,276	10.34	1,441.64	5.14	<0.00001
3	2001-2003	6	229,099	74.57	4,066.36	4.72	<0.00001
4	2006-2007	6	255,928	33.98	2,215.46	3.57	<0.00001
5	2005-2007	14	205,633	90.61	372.02	0.39	<0.00001
6	2003-2005	22	534,977	81.52	266.55	0.28	<0.00001
7	2005-2007	5	125,885	30.30	193.03	0.20	<0.00001
8	2002-2004	13	359,881	68.18	168.94	0.18	<0.00001
9	2005-2007	7	198,563	38.26	129.42	0.13	<0.00001
**10**	2004	45	396,241	91.51	18.14	0.05	<0.00001
**2008-2013**							
1	2010-2011	6	236,132	74,57	2,607.86	3.40	<0.00001
2	2009-2010	43	1,903,505	97,93	128.13	0.15	<0.00001
3	2008-2010	43	1,265,947	98,69	98.97	0.07	<0.00001
4	2009	37	1,284,742	98,87	15.80	0.03	<0.00001
5	2009-2010	9	299,638	95,64	21.35	0,02	<0.00001
**2014-2019**							
1	2019	3	106,663	21.78	2,801.34	9,24	<0.00001
2	2014-2016	2	65,724	38.03	6,799.64	7,57	<0.00001
3	2015	3	39,249	26.29	2,198.78	7,22	<0.00001
4	2015-2017	6	171,946	41.19	3,427.82	3,81	<0.00001
5	2016-2017	15	283,338	90.76	1,782.32	2,95	<0.00001
6	2018-2019	28	774,118	92.58	175.16	0,27	<0.00001
7	2018-2019	24	865,978	85.45	68.59	0,10	<0.00001
8	2017-2019	46	1,555,165	99.40	74.14	0,07	<0.00001

During 2008-2013, five statistically significant clusters were identified. The western region showed a higher RR (RR=3.40) between 2010 and 2011. Low-RR clusters (RR=0.02-0.15) were found to be dispersed in the whole state-cluster 2 in the center-north and northeast; cluster 3 in the northwest; cluster 4 in the south and center-south; and cluster 5 in the east region (Table 1; Figure 2B).

During 2014-2019, eight clusters were identified. The highest RR was identified in the eastern region of Ceará in 2019 (RR=9.24), followed by the western region during 2014-2016 (RR=7.57). There were three other clusters with high RRs-1 in the central area (cluster 3; RR=7.22) in 2015; 1 in the southern area (cluster 4; RR=3.81) during 2015-2017; and 1 in the southeastern area (cluster 5; RR=2.95) during 2016-2017. The clusters with low RRs, similar to the previous periods, were located in the central-north region (cluster 6), central-south and south regions (cluster 7), and northwest region (cluster 8). Clusters 6 and 7 were active during 2018-2019, and cluster 8 was active during 2017-2019. The RRs of these clusters was 0.07-0.27 (Table 1; Figure 2C).

## DISCUSSION

The time series covered in this study comprises more than two-thirds of the years of the occurrence of dengue in Ceará. Time-trend analysis showed a reduction in the incidence coefficients over 19 years, which was more prominent in the last 3 years analyzed (2017-2019). In 2018, although the Northeast region had the highest incidence coefficients in Brazil, Ceará had the third lowest incidence coefficients in the region. Furthermore, there was low transmissions of chikungunya and zika, and only the DENV-1 serotype was reported to be in circulation^21^.

The findings agree with the reduction in the incidence of dengue observed in a cohort in the Southeast region of Brazil during 2014-2018^22^. This decline was also noticed throughout Latin America in 2017, when three hypotheses were considered-modernization of epidemiological surveillance systems, cross-immunity generated by the co-circulation of different arboviruses, and factors related to vector control^23^. 

A panel of experts concluded that the reduction in the incidence of dengue due to changes in the surveillance system was unlikely as the reduction was observed on a large scale and not just in a single country. However, they agreed that part of this reduction in 2017 resulted from the super-notification of cases of zika and chikungunya that circulated silently between 2015 and 2016^23^. 

Furthermore, cross-immunity played a major role in reducing the incidence as patients with a history of dengue who were later infected with Zika virus (ZIKV) have protective immunity against a new dengue infection. However, *in vivo* experiments refute these hypotheses for the Brazilian epidemic^24^ ^-^ ^26^. 

In a previous study, mice pre-immunized for ZIKV and infected with DENV-2 had severe symptoms and lethality until day 5^25^. This suggests that immunity to ZIKV predisposes the mice to increased DENV pathology *in vivo*.

In rhesus monkeys (*Macaca mulatta*), previous infections with ZIKV led to significant increases in DENV-2 viremia accompanied by neutropenia, lymphocytosis, hyperglycemia, and a high number of reticulocytes^26^. With these considerations, group immunity against ZIKV must increase the number of dengue cases in Brazil; however, the dengue cases reduced since the most prevalent serotype at the time was DENV-2^24^.

Otherwise, the hypothesis of cross-reaction with ZIKV could be applied in the state of Ceará based on the history of dengue in the state and because a large part of the population is monotypically immunized. However, despite the population being susceptible to ZIKV since its introduction, the incidence of this arbovirus was not high; there were only 623 confirmed cases during 2017-2019^27^ ^-^ ^29^. Therefore, it is unsure to assume that group immunity for ZIKV and DENV in the population of Ceará would be significant for the reduction of dengue cases.

The probable hypothesis for the reduction in the number of cases in Latin America was the increase and strengthening of vector control interventions, with a strong response from communities regarding the elimination of breeding sites^23^. However, this hypothesis would not be sustained in the Brazilian reality due to the exponential increase in the number of cases of chikungunya in 2016 and 2017, which is also transmitted by the same vector as dengue^24^.

The same reality of Brazil can be applied to the state of Ceará, as in 2017, this state experienced the second and the highest epidemic wave of chikungunya. There were 105,312 confirmed cases, with an incidence of 1,174.9/100,000 inhabitants and 194 deaths. The most important variables of this epidemic were high vector density and susceptibility of the population^30^. There are hypotheses suggesting that even if *Aedes aegypti* are coinfected with chikungunya virus (CHIKV) and DENV, these viral infections do not overlap in humans^31^. If the actions taken to combat the vector was the reason, cases of chikungunya should have reduced in 2017 along with those of dengue.

In Ceará, the reduction in dengue cases over 19 years was probably a consequence of group immunity to DENV. In this state, the four DENV serotypes had already been isolated from suspected dengue cases. Since 1986, DENV-1 has been isolated from suspected cases annually (except for 2004 and 2006-2008). DENV-2 has a history of circulation for >15 years and was isolated in some epidemic years in Ceará (1994, 2001, 2003, and 2006-2008), where there was also a record of a high number of severe dengue cases^7^ ^,^ ^9^.

DENV-3 was introduced in 2002 and was isolated from suspected cases in 2002-2008, 2011, 2014, and 2015. DENV-4 is the most recently introduced serotype and was isolated from suspected cases during 2011-2015^7^ ^,^ ^9^. The reduction in incidence in an endemic region occurs mainly due to group immunity rather than the consequences of control actions^32^. However, failures in the surveillance and notification of cases should be assessed, especially in periods considered to have low transmission.

Space-time analysis of dengue cases revealed clusters of municipalities with high RRs of incidence mainly in the south and west regions of Ceará. In the southern region, during the first period, the high number of cases may be related to vector control policies, such as the mass use of the same organophosphate larvicide since 1986. In 2000, *Ae. aegypti* mosquitoes collected were resistant to this product in some cities, mainly in the southern region and the metropolitan region of Fortaleza (central-north area of the state)^33^, suggesting that chemical control with this product had already lost its effectiveness against *Ae. aegypti*, whose infestation could favor the occurrence of new epidemic outbreaks.

The cluster in the southern region were prevalent in cities with medium-sized and small populations, mainly rural regions^34^. Moreover, it is a strategic place for the transportation of goods and people flow^35^ ^,^ ^36^. This demographic profile demonstrates an important role in maintaining viral circulation at the regional level. A study performed in Southeast Asia identified some areas affected by sequential epidemics; dengue cases from agricultural areas spread to large centers in only 3 months^37^.

The mobility of people between cities and locations increases the dynamics of the spread of dengue, leading to a greater number of infected people^38^ ^,^ ^39^. This reinforces a greater potential for dissemination in cities with a large concentration of industrial, marketing, tourist and academic activities^40^.

In the cluster in the western region of Ceará, it was possible to identify cities that were mostly rural and with low demographic density^34^. Areas with high demographic densities are prone to explosive dengue epidemics due to disordered urbanization^41^. However, cities with low-density demographic conditions also favor the occurrence of dengue. In Paraíba, a state in the Northeast region of Brazil, places with low population density showed a high number of cases due to inadequate disposal of solid waste and inefficiency of the body responsible for the cleaning sector^42^. In Minas Gerais, a state in the Southeast region of Brazil, the coverage of selective waste collection showed an inverse and significant relationship with the incidence of dengue^43^. 

There is a strong complementarity between garbage collection, water supply, and sewage services in a municipality^44^. According to the latest IBGE census, in all municipalities in the western cluster, <40% of the population had sewage services; one municipality had a coverage of only 2% of households^34^. Low-income families are the most affected by the reduced access to basic sanitation services, especially sewage services^44^.

A study in rural areas across Brazil found that municipalities with the lowest gross domestic product *per capita* and the lowest rates of sanitary sewage had the highest risks of dengue^45^. Dengue presents a health challenge in not only urban centers but also peri-urban and rural areas^46^.

It is important to consider that areas with low RRs, such as the northwest, north, and central-north regions of Ceará, had this classification because the neighboring areas had very a high incidence of dengue. In endemic areas, the real incidence dengue is not presented due to underreporting and underdiagnosis^47^. Thus, a hypothesis regarding underreporting and underdiagnoses in these low-risk areas should be discussed.

Underreporting in epidemiological surveillance is a problem generated due to non-notification of asymptomatic or mild cases as people in this situation usually do not seek medical attention, contributing to underreporting^48^ ^,^ ^49^.

Difficulties in accessing health services in the regions studied may have also contributed to the increase in underreporting. Access to health services in Brazil is strongly influenced by the social condition of the people and the place where they live^50^. Among the barriers to accessing health services, the cost of commuting to health centers is the main factor in the Northeast region^51^. People who live far from health facilities tend to seek medical help only in more serious situations^48^. All these factors led to a reduction in the notification of cases when patients are oligosymptomatic for dengue.

The use of secondary data is a limitation of this study given that asymptomatic and oligosymptomatic cases may not avail health service and may not be notified. However, the state of Ceará has one of the best health surveillance systems in Brazil, which reduces this problem. The state has been experiencing a triple burden of arboviruses, including DENV, ZIKV, and CHIKV, which show similar signs and symptoms that influence the differential diagnosis of dengue. Such a situation can lead to possible errors in notifications, but used the official data from the Ministry of Health and the Secretary of Health, with no alternative for this type of study. 

Another limitation occurred in the collection of data on December 9, 2019. The data were not consolidated; however, historically, the last weeks of December had few cases of dengue, and the absence of these data did not influence the results of time-trend and time-space analyses. 

In the process of cluster formation, some municipalities may have been considered to have low RR only due to the proximity of municipalities with high incidence. The results found in each municipality cannot be extrapolated to its subareas. 

In Ceará, dengue incidence coefficients showed a downward trend during 2001-2019, probably reflecting its hyperendemic behavior with the increase in group immunity.

During the epidemic years, there was a relationship between low municipality human development indices and poor socioeconomic conditions, with the presence of high-RR clusters in the southern, eastern, and western regions, thus reinforcing the need for constant surveillance and assistance in these areas.

Even in epidemic years, some municipalities in the north, northwest, central, and central-south regions presented low RRs, which may be related to poor surveillance, as underreporting can hide the real incidences. If these areas actually have low incidences of dengue, it is important to continue surveillance and vector control as the introduction of a new serotype in a more susceptible area could cause explosive outbreaks and a new epidemic in the state.
